# Comparative genomic analyses identify the *Vibrio harveyi* genome sequenced strains BAA-1116 and HY01 as *Vibrio campbellii*

**DOI:** 10.1111/j.1758-2229.2009.00100.x

**Published:** 2010-02

**Authors:** Baochuan Lin, Zheng Wang, Anthony P Malanoski, Elizabeth A O'Grady, Charles F Wimpee, Varaporn Vuddhakul, Nelson Alves Jr, Fabiano L Thompson, Bruno Gomez-Gil, Gary J Vora

**Affiliations:** 1 Center for Bio/Molecular Science & Engineering, Naval Research LaboratoryWashington, DC, USA; 2 Department of Biological Sciences, University of Wisconsin-MilwaukeeMilwaukee, WI, USA; 3 Department of Microbiology, Prince of Songkla UniversityHat Yai, Thailand; 4 Department of Genetics, Federal University of Rio de JaneiroRio de Janeiro, Brazil; 5 CIAD, A.C., Mazatlán Unit for AquacultureSinaloa, Mexico

## Abstract

Three notable members of the Harveyi clade, *Vibrio harveyi*, *Vibrio alginolyticus* and *Vibrio parahaemolyticus*, are best known as marine pathogens of commercial and medical import. In spite of this fact, the discrimination of Harveyi clade members remains difficult due to genetic and phenotypic similarities, and this has led to misidentifications and inaccurate estimations of a species' involvement in certain environments. To begin to understand the underlying genetics that complicate species level discrimination, we compared the genomes of Harveyi clade members isolated from different environments (seawater, shrimp, corals, oysters, finfish, humans) using microarray-based comparative genomic hybridization (CGH) and multilocus sequence analyses (MLSA). Surprisingly, we found that the only two *V. harveyi* strains that have had their genomes sequenced (strains BAA-1116 and HY01) have themselves been misidentified. Instead of belonging to the species *harveyi*, they are actually members of the species *campbellii*. In total, 28% of the strains tested were found to be misidentified and 42% of these appear to comprise a novel species. Taken together, our findings correct a number of species misidentifications while validating the ability of both CGH and MLSA to distinguish closely related members of the Harveyi clade.

## Introduction

The eight *Vibrio* species currently recognized as members of the Harveyi clade (*V. harveyi*, *V. campbellii*, *V. alginolyticus*, *V. rotiferianus*, *V. parahaemolyticus*, *V. natrigens*, *V. mytili* and *V. azureus*) (Sawabe *et al.*, 2007; Yoshizawa *et al.*, 2009) are a subset of the *Vibrio* core group (Reichelt *et al.*, 1976; Dorsch *et al.*, 1992). Members of this clade are commonly found in marine and estuarine surface waters and sediments, as commensals on the surface or within the intestinal flora of marine animals, as opportunistic pathogens, or as primary pathogens of many commercially farmed marine invertebrate and vertebrate species (O'Brien and Sizemore, 1979; Thompson *et al.*, 2004). In addition to thriving in similar environments, members of the Harveyi clade also share a high degree of genetic and phenotypic similarity; so much so that traditional phenotypic identification methods are often unable to confidently identify and differentiate these sister species (Sawabe *et al.*, 2007; Cano-Gomez *et al.*, 2009). For example, *V. harveyi*, *V. campbellii* and *V. rotiferianus*, which form the most recent subclade of speciation within the Harveyi clade (Pascual *et al.*, 2009), have nearly indistinguishable phenotypes (Bryant *et al.*, 1986; Gomez-Gil *et al.*, 2004). These similarities have confounded typing schemes and resulted in documented misidentifications (Gauger and Gomez-Chiarri, 2002; Gomez-Gil *et al.*, 2004). While not exceedingly problematic, these misidentifications do have the potential to overemphasize the importance of a species in a particular setting, especially since most misidentifications are initially characterized as *V. harveyi*.

Considering the economic importance and seemingly continually expanding host range of the Harveyi clade (Austin *et al.*, 2005; Rosenberg *et al.*, 2007; Cervino *et al.*, 2008; Defoirdt *et al.*, 2008), there remains a continued interest in the development of methods to identify and differentiate its members. In contrast with phenotypic identification methods, two genetic methods, DNA–DNA hybridization (DDH) and multilocus sequence analysis (MLSA), have successfully been applied to the study of *Vibrio* taxonomy and evolutionary history (Reichelt *et al.*, 1976; Gomez-Gil *et al.*, 2003; 2004; Thompson *et al.*, 2005; 2007; 2008; Sawabe *et al.*, 2007; Pascual *et al.*, 2009). DDH in particular has been accepted for decades as the standard method for species delineation as it enables a direct assessment of overall genetic similarity and grouping by comparing the extent to which two genomes hybridize to one another (Gevers *et al.*, 2005). Comparative genomic hybridization (CGH) using whole genome microarrays relies upon the same biophysical properties and can be considered a natural technological extension of DDH. However, CGH analyses also offer the added benefit of coding sequence (CDS) level resolution thus providing a greater number of data points that can be used to simultaneously evaluate clade or species-level genetic diversity and environment-specific genetic assemblages. Thus, CGH analyses provide high information content and discriminatory power in a format that is amenable to archiving in electronic databases for future strain comparisons (Gevers *et al.*, 2005; Sawabe *et al.*, 2007; Cano-Gomez *et al.*, 2009). In this study, we employed CGH using a custom-designed Affymetrix *V. harveyi* BAA-1116/HY01 DNA microarray to delineate 38 geographically, environmentally and temporally distributed members of the Harveyi clade and confirmed the resulting cluster assignments using two MLSAs.

## Results and discussion

### CGH analysis

A total of 43 previously characterized isolates (29 *V. harveyi*, seven *V. campbellii*, two *V. parahaemolyticus*, three *V. rotiferianus* and two *V. alginolyticus*), from a wide temporal, geographical and environmental distribution were selected for this study (Table 1). A subset of 38 isolates were analysed via CGH using a custom-designed Affymetrix DNA microarray (Vharveyi520694F) that targets 4831 total CDS from the fully assembled and annotated *V. harveyi* BAA-1116 genome (Naval Research Laboratory sequencing effort GenBank CP001223-5) and 965 CDS unique to the unfinished *V. harveyi* HY01 genome sequence (GenBank AAWP00000000). The targeted CDS did not include insertion sequence elements, transposons or repeat sequences as they were omitted from the microarray design.

**Table 1:** Geographically and temporally distributed Harveyi clade strains used in this study.

Strain	Assigned species	Origin	Location/year	Source	Used in
CAIM 29	*harveyi*	Diseased shrimp (*Litopenaeus* sp.) larvae isolate	Jepara, Indonesia/1990	CAIM	CGH/MLSA
CAIM 148	*harveyi*	Diseased shrimp (*Penaeus* sp.) haemolymph isolate	Valle de Matatipac, Mexico/1995	CAIM	CGH/MLSA
CAIM 461	*harveyi*	Shark tank water isolate	Denmark/1993	CAIM	CGH/MLSA
CAIM 463	*harveyi*	Sea bass (*Dicentrarchus labrax*) isolate	Greece/1991	CAIM	CGH/MLSA
CAIM 464	*harveyi*	Turbot (*Scophthalmus maximus*) isolate	Spain/1990	CAIM	CGH/MLSA
CAIM 606^a^	*harveyi*	Japanese horse mackerel (*Trachurus japonicus*) isolate	Uchiura Bay, Numazu, Japan/1992	CAIM	CGH/MLSA
CAIM 1075	*harveyi*	Oyster (*Crassostrea gigas*) isolate	SCPA Burabampo SCL, Mexico/2003	CAIM	CGH/MLSA
CAIM 1754	*harveyi*	Puffer fish (*Spheroides annulatus*) heart isolate	CIAD AC, Mexico/2005	CAIM	CGH/MLSA
CAIM 1766	*harveyi*	Sea horse (*Hipocampus ingens*) liver isolate	Mazatlan Aquarium, Mexico/2005	CAIM	CGH/MLSA
CAIM 1792	*harveyi*	Diseased shrimp (*Litopenaeus vannamei*) lesion isolate	Aquastrat S.A. de C.V., Mexico/2005	CAIM	CGH/MLSA
ATCC BAA-1116^f^	*harveyi*	Marine (ocean) isolate	Unknown/1993	ATCC	CGH/MLSA
ATCC 33843	*harveyi*	392 [MAV]	Woods Hole, USA/1971	ATCC	CGH/MLSA
ATCC 35804^b^	*harveyi*	Brown shark (*Carcharhinus plumbeus*) kidney isolate	Baltimore, USA/1982	ATCC	CGH/MLSA
ATCC 14126^c^	*harveyi*	Dead, luminescing amphipod (*Talorchestia* sp.) isolate	Woods Hole, USA/1935	ATCC	CGH/MLSA
D1	*harveyi*	White grunt (*Haemulon plumieri*) gut isolate	Chub Cay, Bahamas/1992	UWM	CGH/MLSA
E1	*harveyi*	White grunt (*H. plumieri*) gut isolate	Chub Cay, Bahamas/1992	UWM	CGH/MLSA
G1	*harveyi*	White grunt (*H. plumieri*) gut isolate	Chub Cay, Bahamas/1992	UWM	CGH/MLSA
H6	*harveyi*	Squirrelfish (*Holocentrus* sp.) gut isolate	Chub Cay, Bahamas/1992	UWM	CGH/MLSA
L1	*harveyi*	White grunt (*H. plumieri*) gut isolate	Chub Cay, Bahamas/1992	UWM	CGH/MLSA
501J	*harveyi*	Surface water isolate	Boca Ciega Bay, Florida, USA/2001	UWM	CGH/MLSA
602L	*harveyi*	Surface water isolate	Boca Ciega Bay, Florida, USA/2002	UWM	CGH/MLSA
9567-98	*harveyi*	Research isolate	Unknown/1998	CDC	CGH/MLSA
2415-05	*harveyi*	Human blood isolate	Hawaii, USA/2005	CDC	CGH/MLSA
74F	*harveyi*	Diseased coral (*Mussimilia braziliensis*) isolate	Abrolhos bank, Brazil/2007	UFRJ	CGH/MLSA
PA2	*harveyi*	Diseased coral (*M. braziliensis*) isolate	Abrolhos bank, Brazil/2007	UFRJ	CGH/MLSA
1DA3	*harveyi*	Diseased coral (*Phyllogorgia dilatata*) isolate	Abrolhos bank, Brazil/2007	UFRJ	CGH/MLSA
50A	*harveyi*	Healthy coral (*Mussimilia hispida*) isolate	Abrolhos bank, Brazil/2007	UFRJ	CGH
9078-83	*harveyi*	Research isolate	Unknown/1983	CDC	CGH/MLSA
HY01	*harveyi*	Dead, luminescing shrimp isolate	Hat Yai, Thailand/2004	PSU	CGH/MLSA
CAIM 115	*campbellii*	Shrimp (*Litopenaeus* sp.) haemolymph isolate	Mexico/1999	CAIM	CGH/MLSA
CAIM 198	*campbellii*	Shrimp (*Litopenaeus* sp.) hepatopancreas isolate	Sinaloa, Mexico/1999	CAIM	CGH/MLSA
CAIM 519T^d^	*campbellii*	Seawater isolate	Hawaii, USA/2005	CAIM	CGH/MLSA
CAIM 1500	*campbellii*	Snapper (*Lutjanus guttatus*) liver isolate	Sinaloa, Mexico/1972	CAIM	CGH/MLSA
2SA4	*campbellii*	Healthy coral (*P. dilatata*) isolate	Abrolhos bank, Brazil/2007	UFRJ	CGH/MLSA
42A	*campbellii*	Healthy coral (*M. hispida*) isolate	Abrolhos bank, Brazil/2007	UFRJ	CGH/MLSA
AND4	*campbellii*	Surface water isolate	Andaman Sea, Indonesia/2000	GenBank	MLSA
CAIM 577T^e^	*rotiferianus*	Rotifer (*Brachionus plicatilis*) water isolate	Ghent, Belgium/1999	CAIM	MLSA
CAIM 994	*rotiferianus*	Snapper (*L. guttatus*) kidney isolate	Sinaloa, Mexico/2004	CAIM	CGH/MLSA
1975	*rotiferianus*	Snapper (*Pagrus auratus*) isolate	Unknown/1999	UFRJ	MLSA
F5828	*parahaemolyticus*	O3:K6 human isolate	Texas, USA/1998	CDC	CGH/MLSA
RIMD2210633	*parahaemolyticus*	O3:K6 human isolate	Osaka, Japan/1996	GenBank	MLSA
40B	*alginolyticus*	Diseased coral (*M. braziliensis*) isolate	Abrolhos bank, Brazil/2007	UFRJ	CGH/MLSA
R-1249	*alginolyticus*	Coral (*Fungia* sp.) yellow band lesion isolate	Sulawesi, Indonesia/2004	PU	MLSA

a Type strain. *Vibrio trachuri* is a junior synonym of *V. harveyi* (Thompson *et al.*, 2002).

b Type strain. *Vibrio carchariae* is a junior synonym of *V. harveyi* (Pedersen *et al.*, 1998).

c Type strain. *Vibrio harveyi*.

d Type strain. *Vibrio campbellii* ATCC 25920.

e Type strain. *Vibrio rotiferianus*.

f Also known as strain BB120 (Bassler *et al.*, 1997).

CAIM, Collection of Aquatic Important Microorganisms (http://www.ciad.mx/caim); ATCC, American Type Culture Collection (http://www.atcc.org); UWM, University of Wisconsin-Milwaukee; CDC, Centers for Disease Control and Prevention, USA; UFRJ, Federal University of Rio de Janeiro; PSA, Prince of Songkla University; PU, Pace University.

Sample preparation for microarray hybridization was performed by extracting, fragmenting and biotin-labelling 3 µg of genomic DNA from each strain according to Affymetrix standard protocols. Biotinylated material was hybridized to the Vharveyi520694F microarrays for 16 h at 49°C in a GeneChip® Hybridization Oven 640 at 60 r.p.m. The microarrays were subsequently washed and stained using the GeneChip® Fluidics Station 450 and scanned using the GeneChip® Scanner 7G. All hybridization signal intensities were analysed with the GeneChip® Operating Software (GCOS) to generate raw image files (.DAT) and summary data files (.CEL). The Bioconductor/R ‘ReadAffy’ and ‘expresso’ functions were used to perform RMA background corrections and CDS summarizations using the avgdiff and MAS methods (Gentleman *et al.*, 2004). No microarray normalization was applied. The results of both summarization methods did not differ significantly so only the avgdiff results are described. The CDS hybridization intensities of each microarray varied from 0 to 14 in log2 representations and were divided into 250 bins of width 0.0056. The number of CDS that fell into each bin was counted and plotted versus intensity. These plots were examined for the presence of two peaks for each microarray as it was expected that the majority of intensities observed for each CDS should form two clusters (present or absent). Every sample, with the exception of CAIM 29, had indications of two peaks and the data points in the immediate region of each peak were fit to a Gaussian function. The fitted centre of each peak and sigma of the Gaussian function were then used to determine cut-off values. All CDS intensities below the smaller centre +2× the sigma value of that peak (siglow) were considered ‘absent’. All CDS intensities greater than the larger centre −2× the sigma value of that peak (sighigh) were considered ‘present’. CDS intensity values between these values were considered ‘uncertain’. The ‘uncertain’ calls were further subdivided into three groups (‘intermediate low’, ‘intermediate’ and ‘intermediate high’). The ‘intermediate low’ region was defined as between +2× and +4× siglow of the low intensity peak. The ‘intermediate high’ region was defined as between −2× and −4× sighigh of the high intensity peak. The ‘intermediate’ region was defined by values that fell between the low intensity peak +4× siglow and the high intensity peak −4× sighigh.

**Fig. 1:**
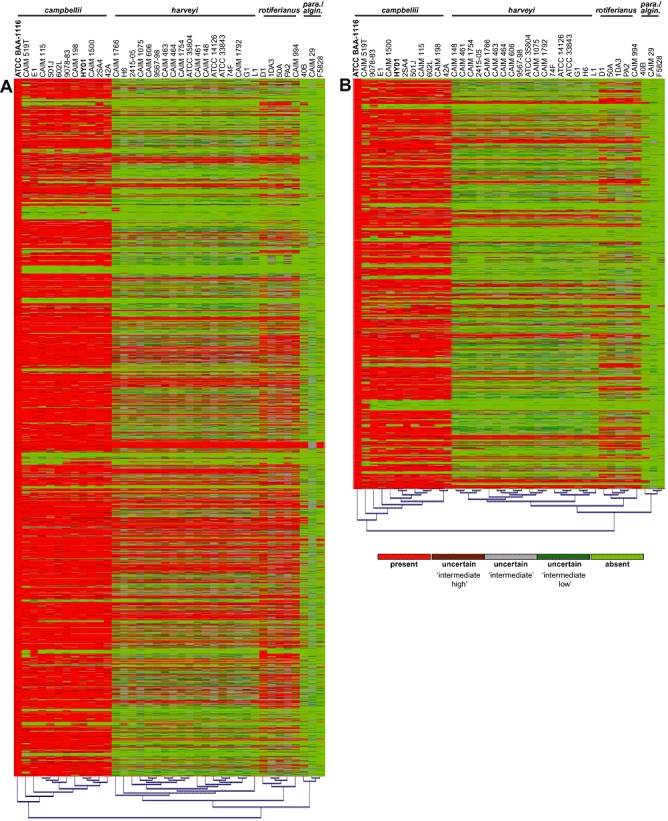
Hierarchically clustered heat maps based on CGH profiles demonstrating the presence and absence of genes within Harveyi clade members with respect to *V. harveyi* BAA-1116. A. Chromosome I, 2999 CDS. B. Chromosome II, 1765 CDS. The CDS in each heat map are ordered according to the genome structure of strain BAA-1116. Each CDS is depicted by one of five possible hybridization states (scale bar): (i) positive hybridization (CDS present call) = bright red bars, (ii) between positive and intermediate hybridization (uncertain ‘intermediate high’ call) = dark red bars, (iii) intermediate hybridization (uncertain ‘intermediate’ call) = grey bars, (iv) between intermediate and no hybridization (uncertain ‘intermediate low’ call) = dark green bars, and (v) no hybridization (CDS absent call) = bright green bars. Presence/absence designations generated from the hybridization profiles were calculated using the avgdiff method and clustered and visualized using MultiExperiment Viewer (MeV v4.4) software. ‘Para./algin.’ = *parahaemolyticus* and *alginolyticus* subclade.

Comparative genomic hybridization profiles were visualized with hierarchically clustered heat maps using the empirical hybridization data from *V. harveyi* BAA-1116 as the strain comparison outgroup (Fig. 1). The aggregate hybridization states (present, three uncertain states, or absent) of 4764 CDS from chromosomes I and II divided the 38 tested strains into four distinct subclades: the *campbellii* subclade which harboured the *V. campbellii* type strain CAIM 519T (ATCC 25920), the *harveyi* subclade which harboured the *V. harveyi* type strain ATCC 14126, the *rotiferianus* subclade and the *parahaemolyticus*/*alginolyticus* subclade (Fig. 1A and B). Interestingly, the hierarchical cluster analyses from both chromosomes placed six purported *V. harveyi* strains (BAA-1116, E1, 501J, 602L, 9078-83 and HY01) in the *campbellii* subclade along with the six tested *V. campbellii* strains. Similarly, *V. harveyi* strain CAIM 29 was located in the *parahaemolyticus*/*alginolyticus* subclade instead of within the *harveyi* subclade and *V. harveyi* strains D1, PA2, 1DA3 and 50A formed a distinct subclade with the sole *V. rotiferianus* strain (CAIM 994) tested in this analysis. Notably, these same four subclades were observed when a hierarchically clustered heat map was generated by comparing each of the strains to the 965 CDS unique to strain HY01 and absent from strain BAA-1116 (data not shown).

Caution is often advised when molecular identification or phylogenetic methods result in novel or unanticipated groupings as genetic recombination may have occurred among related species thus confounding the results. This is an especially significant consideration when using too few molecular markers or when dealing with the genetically dynamic vibrios, as it is well accepted that recombination and mobile genetic elements have played a critical role in the evolution of the genus (Faruque and Mekalanos, 2003; Thompson *et al.*, 2004; Sawabe *et al.*, 2007; Pascual *et al.*, 2009). However, as the hybridization state of each CDS can be considered a unique and independent data point, the scale of the CGH analysis efficiently neutralizes the effect of small-scale recombination, point mutations, horizontal gene transfer and overall genetic plasticity with respect to gene content and primary sequence identity. Thus, the CGH analyses strongly suggested that although previously characterized as *V. harveyi*: (i) strains E1, 501J, 602L, 9078-83 and the genome sequenced strains BAA-1116 and HY01 belong to the species *campbellii*, (ii) strain CAIM 29 belongs to the species *parahaemolyticus*, and (iii) strains D1, PA2, 1DA3 and 50A form a distinct subclade with *V. rotiferianus* CAIM 994.

### Harveyi clade MLSAs

**Fig. 2:**
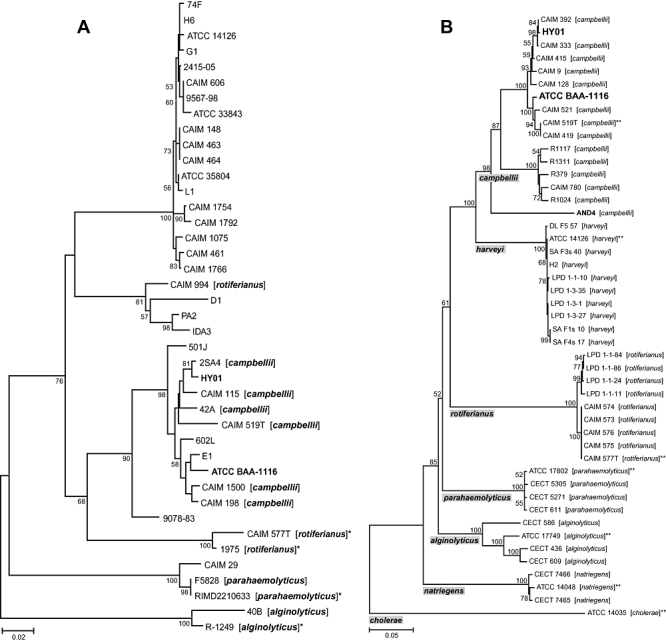
Multilocus sequence analysis (MLSA) of Harveyi clade members. A. Phylogenetic tree based on the Neighbour-Joining method using concatenated sequences from the *ftsZ*, *mreB* and *topA* genes (1528 nt) and MEGA software v4.0. Original species designations are in brackets. Strains lacking species designations were originally identified as *V. harveyi*. This analysis includes all of the strains used in the CGH analyses (with the exception of strain 50A) and four additional strains that are denoted with an asterisk ‘*’. The primary sequence information has been submitted to the GenBank database and the relevant accession numbers can be found in Table S1. B. Phylogenetic tree based on the Neighbour-Joining method using concatenated sequences from the *rpoD*, *rctB* and *toxR* genes (1848 nt) and MEGA software v4.0. Strain identifiers ending in ‘**’ denote type strains. With the exception of BAA-1116, HY01 and AND4 (bold type), all sequences used in this MLSA were downloaded from the ‘Taxonomy of the Vibrios’ database (http://www.taxvibrio.lncc.br/). Alignments for both analyses were generated using the clustalw program and bootstrap percentages > 50% from 1000 simulations are shown to the left of each branch point. The scale bar represents the number of substitutions per site.

Multilocus sequence analysis is a sequence-based genotypic characterization method that has successfully been used to establish species-level taxonomy within the Harveyi clade (Thompson *et al.*, 2007; Pascual *et al.*, 2009). To validate the subclade designations generated by our CGH analyses and further solidify species assignments, we subjected the same panel of strains used in the CGH analyses to a previously validated three-gene MLSA scheme used for the classification of core *Vibrio* species (Thompson *et al.*, 2007). The *ftsZ* (cell division protein), *mreB* (rod shape-determining protein) and *topA* (topoisomerase I) genes were PCR amplified as previously described (Thompson *et al.*, 2007) to generate products for sequencing and the resulting sequences were concatenated (1528 nt) and subjected to phylogenetic analysis based on the Neighbour-Joining method. Sequences from four additional strains with confirmed species identities [*V. rotiferianus* 1975 and CAIM 577T (type strain) (http://www.taxvibrio.lncc.br/), *V. parahaemolyticus* RIMD 2210633 (GenBank BA000031-2) and *V. alginolyticus* R-1249 (Cervino *et al.*, 2008)] were also added to strengthen the analysis. The resulting phylogeny revealed that the *ftsZ*-*mreB-topA* MLSA-derived major subclade designations were nearly identical to those seen in the CGH analyses and strains BAA-1116 and HY01 were once again found nested within the *campbellii* subclade (Fig. 2A). While *V. harveyi* and *V. campbellii* both formed monophyletic subclades, *V. rotiferianus* did not. Interestingly, the added *V. rotiferianus* strains (type strain CAIM 577T and 1975) did not group with strains CAIM 994, D1, PA2 and 1DA3 which were designated as the ‘*rotiferianus* subclade’ (Fig. 1) based on the original identification of strain CAIM 994 (Table 1). Rather, strains CAIM 994, D1, PA2 and 1DA3 formed a unique cluster that appeared to be most closely related to the *harveyi* subclade. A comparison of the concatenated sequence % identity found the members of this cluster to be 91.5–94.1% identical to the *V. harveyi* type strain (ATCC 14126) and 90.4–91.8% identical to the *V. rotiferianus* type strain (CAIM 577T). Strain 50A, which was also considered a member of the ‘*rotiferianus* subclade’ based on the CGH analyses, was omitted from the MLSA as we were unable to amplify its *ftsZ* gene using the previously described VftsZ75F/VftsZ800R primer pair and amplification method (Sawabe *et al.*, 2007). Nevertheless, the use of a truncated concatenated sequence (*mreB* and *topA* only) strongly grouped strain 50A with strains CAIM 994, D1, PA2 and 1DA3 (98% bootstrap support, data not shown). Thus, the formation of this unique subclade, to the exclusion of the *V. rotiferianus* type strain, and its position relative to the *harveyi* and *campbellii* subclades suggests that CAIM 994 has been misidentified as *V. rotiferianus* and that strains CAIM 994, D1, PA2, 1DA3 and 50A likely denote a novel species within the Harveyi clade.

As it is acknowledged that recombination may have occurred in some of the loci used in the *ftsZ*-*mreB-topA* MLSA (Thompson *et al.*, 2007), we sought an additional confirmation of the derived classifications of the genome sequenced strains using three independent markers. The *rpoD* (RNA polymerase, sigma 70 factor), *rctB* (replication initiator protein) and *toxR* (virulence regulatory protein) gene sequences from strains BAA-1116 and HY01 and the genome sequenced strain *V. campbellii* AND4 (GenBank ABGR00000000) were concatenated (1848 nt), aligned with 44 concatenated sequences utilized in a MLSA by Pascual and colleagues (2009), and subjected to phylogenetic analysis using the Neighbour-Joining method. The resulting phylogeny, which included the type strain of each of the seven species tested, verified the *V. campbellii* classification of strains BAA-1116 and HY01 and parsed each species as a monophyletic subclade (Fig. 2B). Thus, the use of a second MLSA scheme with an entirely different set of Harveyi clade members with confirmed species designations (Pascual *et al.*, 2009) corroborated the *ftsZ*-*mreB-topA* MLSA and CGH analyses findings that strains BAA-1116 and HY01 belong to the species *campbellii*.

### CGH- and MLSA-based observations

*In toto*, our findings support three salient observations. First, of the 43 Harveyi clade members tested in this study, 12 (28%) appear to have been misidentified: five of which appear to represent a novel species. To some extent the misidentifications were to be expected as distinguishing members of the Harveyi clade is known to be a difficult taxonomic task (Sawabe *et al.*, 2007) and the advent of genetic methods with high discriminatory power has previously elucidated misidentifications in a substantial percentage of strains tested [71% (Gomez-Gil *et al.*, 2004) and 18% (Pascual *et al.*, 2009)]. Considering the relatively recent estimated radiation time of 39 million years for *V. harveyi* and *V. campbellii* (Sawabe *et al.*, 2007) and previous findings (Gomez-Gil *et al.*, 2004), it was not surprising that half of misidentifications revealed in this study were *V. campbellii* mistaken as *V. harveyi*. What was clearly surprising is that the two purported *V. harveyi* strains that have had their genomes sequenced (strains BAA-1116 and HY01) have themselves been misidentified. The frequent misidentification of *V. campbellii* as *V. harveyi* has led to the assertion that *V. campbellii* is currently underestimated as an important pathogenic species of aquatic organisms (Gomez-Gil *et al.*, 2004; Cano-Gomez *et al.*, 2009). Our findings with strain BAA-1116, and more importantly strain HY01, which is known to be a serious shrimp pathogen (Rattanama *et al.*, 2009), provide additional evidence to support this assertion.

Second, analysis of the CGH results indicated that 72–77% of the CDS from BAA-1116 chromosomes I and II were considered present in the *campbellii* subclade strains (9078-83, 501J, 602L, 2SA4, HY01, CAIM 1500, CAIM 198, CAIM 115, E1, CAIM 519T, 42A). This percent similarity is in agreement with DDH findings that have shown the intraspecies percentage similarity for *V. campbellii* strains to be 71–80%. This is markedly less than the 96–100% intraspecies similarity seen for *V. harveyi* strains (Pascual *et al.*, 2009) suggesting that *V. campbellii* is more genetically diverse than *V. harveyi*. Fluorescent amplified fragment length polymorphism (FAFLP) analyses bolster this contention as they have previously revealed that the *V. campbellii* group is very diverse (FAFLP value < 10%), much more so than *V. harveyi* and *V. rotiferianus* (FAFLP value ≥ 45%) (Thompson *et al.*, 2001; Gomez-Gil *et al.*, 2004). In addition, both *ftsZ*-*mreB-topA* and *rpoD*-*rctB*-*toxR* MLSA phylogenies reveal longer branch lengths within the *campbellii* subclade than the *harveyi* subclade (Fig. 2A and B) signifying a greater genetic distance and enhanced rate of evolution within *V. campbellii*. Taken together, the genetic data indicate that *V. campbellii* is evolving at a faster rate and thus more genetically heterogeneous than *V. harveyi*.

Finally, although autoinduction was first described in *V. harveyi* using strain 392 [MAV] (ATCC 33843) (Nealson *et al.*, 1970; Baumann *et al.*, 1980) [previously described as MAV (Hastings *et al.*, 1969), *Photobacterium fischeri* strain MAV (Nealson *et al.*, 1970) and *Beneckea harveyi* strain 392 (Reichelt and Baumann, 1973)], the molecular mechanisms of *V. harveyi* quorum sensing have been most extensively studied in strain BAA-1116 [also known as strain BB120 (Bassler *et al.*, 1997)] and it has consequently become a model system for quorum sensing research (Bassler *et al.*, 1997; Henke and Bassler, 2004; Lenz *et al.*, 2004; Waters and Bassler, 2006; Tu and Bassler, 2007; Long *et al.*, 2009). However, as our findings identify strain BAA-1116 as *V. campbellii* and not *V. harveyi* and the quorum sensing architectures within the genus are known to be varied (Milton, 2006), it will be interesting to see how similar the *V. harveyi* quorum sensing system is to the well-described BAA-1116 quorum sensing system.

### Concluding remarks

In this study, we have highlighted the ongoing difficulty of accurately identifying closely related *Vibrio* core group members. When considering the Harveyi clade, the results of this study and others suggest that a re-evaluation of the genetic or phenotypic markers commonly used to discriminate these species is needed. Comparative genomic hybridization analyses can contribute to this effort by distinguishing unique genus, species and strain-specific genetic targets for molecular identification methods development. The continued analysis of this data set to find such genetic targets, establish the *V. campbellii* core genome and potentially reveal the underlying genetic assemblages responsible for observed pathogenic or niche adaptation phenotypes is ongoing. Although there exists a large body of literature pertaining to the study of *V. harveyi* ATCC strain BAA-1116 (BB120), especially with respect to quorum sensing, these findings necessitate a change in species designation. The genome sequenced strains *V. harveyi* BAA-1116 and *V. harveyi* HY01 should hereafter be properly identified as *V. campbellii* BAA-1116 and *V. campbellii* HY01. By extension, the results also indicate that we now lack a representative genome sequence from the namesake of the Harveyi clade. *Vibrio harveyi* is a species that has been central to our understanding of bacterial bioluminescence and quorum sensing and continues to be a formidable pathogen in the aquaculture industry. As such, a *V. harveyi* genome sequencing effort is warranted.

## Supporting information

Table S1. Accession numbers and source of MLSA sequences used in this study.
